# Quantitative Description of Glycan-Receptor Binding of Influenza A Virus H7 Hemagglutinin

**DOI:** 10.1371/journal.pone.0049597

**Published:** 2013-02-20

**Authors:** Karunya Srinivasan, Rahul Raman, Akila Jayaraman, Karthik Viswanathan, Ram Sasisekharan

**Affiliations:** Harvard-MIT Division of Health Sciences and Technology, Koch Institute for Integrative Cancer Research, Singapore-MIT Alliance for Research and Technology, Department of Biological Engineering, Massachusetts Institute of Technology (MIT), Cambridge, Massachusetts, United States of America; University of Ottawa, Canada

## Abstract

In the context of recently emerged novel influenza strains through reassortment, avian influenza subtypes such as H5N1, H7N7, H7N2, H7N3 and H9N2 pose a constant threat in terms of their adaptation to the human host. Among these subtypes, it was recently demonstrated that mutations in H5 and H9 hemagglutinin (HA) in the context of lab-generated reassorted viruses conferred aerosol transmissibility in ferrets (a property shared by human adapted viruses). We previously demonstrated that the quantitative binding affinity of HA to α2→6 sialylated glycans (human receptors) is one of the important factors governing human adaptation of HA. Although the H7 subtype has infected humans causing varied clinical outcomes from mild conjunctivitis to severe respiratory illnesses, it is not clear where the HA of these subtypes stand in regard to human adaptation since its binding affinity to glycan receptors has not yet been quantified. In this study, we have quantitatively characterized the glycan receptor-binding specificity of HAs from representative strains of Eurasian (H7N7) and North American (H7N2) lineages that have caused human infection. Furthermore, we have demonstrated for the first time that two specific mutations; Gln226→Leu and Gly228→Ser in glycan receptor-binding site of H7 HA substantially increase its binding affinity to human receptor. Our findings contribute to a framework for monitoring the evolution of H7 HA to be able to adapt to human host.

## Introduction

Avian influenza virus subtypes known to infect and cause disease in humans include H5N1, H7N7, H7N2, H7N3 and H9N2 strains. These viruses circulate in domestic poultry but have not yet adapted to the human host to establish sustained airborne human-to-human transmission capabilities [1]. One of the characteristic features of human-adapted subtypes such as H1N1, H2N2 and H3N2 is the ability of their viral surface glycoprotein hemagglutinin (HA) to bind preferentially to α2→6 sialylated glycan receptors (or *human receptors*) that are predominantly expressed in the human upper respiratory epithelium. The HA of influenza viruses isolated from avian species typically binds to α2→3 sialylated glycans (or *avian receptors*) [2]. Therefore, the gain in the ability of HA from an avian isolate (such as H5, H7, H9, etc.) to preferentially bind to human receptors (high relative binding affinity to human receptor over avian receptor) is implicated as one of the important factors for the human adaptation of the virus [3]. In the past few years, novel influenza strains such as 2009 H1N1 and 2010 H3N2 that naturally emerged from multiple reassortment of viral gene segments between avian, swine and human isolates were able to successfully adapt to human host [4], [5]. In the context of these novel strains, the avian influenza subtypes pose a significant threat of human adaptation [1]. With the human population predominantly naïve to these avian influenza antigens, constant surveillance with particular focus on molecular changes geared towards human host adaptation becomes vital in this era of pandemics [6].

Specific mutations in glycan-receptor binding site (RBS) of H5 and H9 HAs have been shown to correlate with respiratory droplet transmissibility of laboratory-generated reassorted viruses possessing either of these mutant HAs (and internal genes from human-adapted virus) in a ferret animal model [7]–[10]. Aerosol transmissibility in ferrets, a hallmark property of human-adapted viruses, has been shown to correlate with specificity and quantitative affinity of viral HA binding to human receptors [11]–[13]. In fact a single amino acid mutation Gln226→Leu in H2 HA completely shifts its receptor binding preference from avian to human receptors and confers airborne viral transmission in ferrets [14]. Studies on H7 subtype have thus far focused on specific H7N7 and H7N2 strains isolated from infected patients in Eurasia and North America respectively (**Figure S1**). The H7N7 strains were isolated from two patients with very different clinical conditions during a local highly pathogenic outbreak in the Netherlands in 2003 [15]. One of the strains was isolated from a patient with a conjunctivitis infection (A/Netherlands/230/03 referred to henceforth as CC); which is typical of H7 human infection, and the other was isolated from a patient with acute respiratory illness that eventually resulted in fatality (A/Netherlands/219/03 henceforth referred to as FC); which was the first of its kind. The HAs from both CC and FC comprise of the polybasic sequence between HA1 and HA2 analogous to the highly pathogenic H5N1. The RBS of CC and FC HAs differs by a single amino acid substitution in position 135 (H3 numbering), which is Ala in CC but Thr in FC (**Figure S1**). The presence of Thr in 135 in FC introduces a glycosylation sequon at Asn-133 [16]–[18].

The H7N2 strain A/New York/107/03 or NY/107 was isolated from a single human case with respiratory infection [15]. The NY/107 HA does not possess the HA1-HA2 polybasic sequence, which is typically associated with high pathogenicity. A dramatically unique feature of NY/107 HA is the complete deletion of the 220-loop region in the RBS (**Figure S1**), which plays a key role in governing glycan receptor-binding specificity of HA [19].

The glycan receptor-binding properties of CC, FC and NY/107 HAS have been characterized by screening the HAs and whole viruses on glycan array platform [20]–[23]. These screening assays provide an overall readout in terms of the number and different types of avian and human receptors that bind to the HA (or virus) when analyzed at a high protein concentration or virus titer. A limitation of such screening studies is that they do not quantify the relative binding affinities of HA to avian versus human receptor. It is important to quantify the nuances in relative binding affinities in order to understand how molecular changes in the HA such as deletion of 220-loop and differences in glycosylation at Asn-133 impinge on glycan receptor binding. We have previously demonstrated that a change in glycosylation at a single site subtly alters human receptor-binding affinity of the pandemic 1918 H1N1 HA [24]. Furthermore we have demonstrated that quantitative parameters derived from a dose-dependent binding of HA to representative human and avian receptors correlated with the respiratory droplet transmissibility of the virus [11]–[13].

In this study, we quantify the relative human and avian receptor-binding affinity of CC, FC and NY/107 HA. We also characterized the effect of altering glycosylation at Asn-133 on NY/107 HA in the context of the deletion of 220-loop on the relative glycan-receptor binding affinities of this HA. Finally we introduced the Gln226→Leu and Gly228→Ser double mutation in the RBS of FC and CC HAs since the Leu-226/Ser-228 combination in the RBS is a hallmark of all the human-adapted H3N2 HAs (belong to the same group 2 clade as H7) and characterize the effect of these mutations on quantitative glycan receptor-binding affinity of these HAs. Our study provides important biochemical insights for monitoring the evolution of H7 HAs as they continue to circulate in avian species and cause sporadic human outbreaks and also pose a constant threat of adapting to human host through reassortment.

## Results

FC, CC and NY/107 HA were recombinantly expressed as described earlier [12], [13]. Given that FC and CC HA differ by a single amino acid change at RBS, CC HA was generated by introducing a Thr135→Ala mutation through site-directed mutagenesis. The wild type and mutant HAs were analyzed on a glycan array platform in a dose-dependent fashion and an apparent binding parameter Kd' was calculated to quantify the relative binding affinities as described earlier [12], [13] (see Methods).

### Quantitative glycan-receptor binding affinities of H7N7 CC and FC HAs

**Figure 1:**
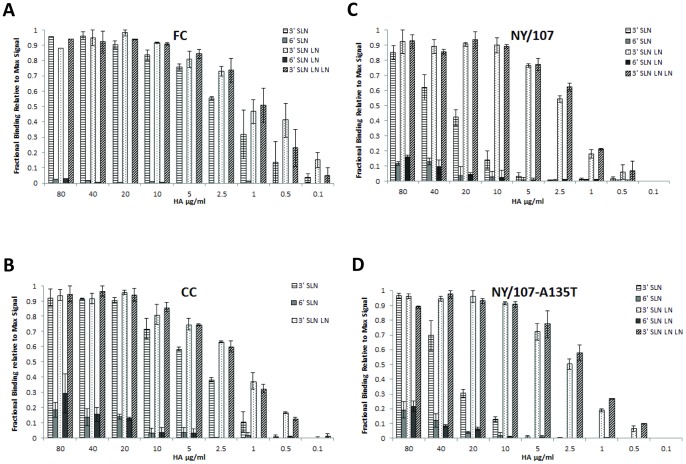
Glycan receptor-binding specificity of FC, CC, NY/107 and mNY/107:A135T HA. **A**, shows dose-dependent direct glycan array binding of FC HA. Specific high affinity binding to avian receptors (3′ SLN, 3′ SLNLN and 3′ SLNLNLN) and no binding to human receptors is observed. **B**, shows dose-dependent direct glycan array binding of CC HA. High affinity binding to avian receptors is observed. In comparison with FC, there is observable binding to human receptors (6′SLN-LN and 6′SLN) albeit at orders of magnitude lower affinity than binding to avian receptors. **C**, shows dose-dependent direct glycan array binding of NY/107 HA. High affinity binding to avian receptors (3′SLN-LN and 3′SLN-LN-LN) is observed with binding affinity for 3′SLN lower than that of FC and CC HAs. Binding to human receptor is observed but at much lower affinity (by orders of magnitude) than binding to avian receptors. **D**, shows dose-dependent direct glycan array binding of mutant mNY/107:A135T HA. Introduction of glycosylation sequon at Asn-133 does not seem to alter binding of this mutant HA in relation to the wild type.

FC HA exclusively bound to the avian receptors, 3′ SLN, 3′SLN-LN and 3′ SLN-LN-LN (Kd' ∼25 pM; based on 3′SLN-LN and 3′SLN-LN-LN; Kd values were similar for binding for both glycans) (Figure 1A). The apparent binding affinity of FC HA to avian receptors was comparable to HAs from avian H2N2 and H5N1 strains analyzed in a similar fashion previously [12], [13]. The HA from CC which lacks glycosylation at Asn-133 also showed predominant binding to avian receptors (Kd'∼65 pM) (based on 3′ SLN-LN 3′SLN-LN-LN binding; Kd values were similar for binding for both glycans) (Figure 1B). CC showed observable binding to human receptors in a dose dependent fashion although at several orders of magnitude lower than avian receptor binding (Kd' was not calculated since saturation was not reached in the concentration window for avian receptor binding). The presence of glycosylation at Asn-133 therefore appears to increase avian receptor specificity for H7N7 HAs. On the other hand lack of glycosylation at this site appears to increase propensity for binding to human receptors.

### Quantitative glycan-receptor binding affinities of H7N2 NY/107 HA

As mentioned earlier, NY/107 HA has a deletion of 8 amino acids in the 220-loop (which is almost the entire loop). The glycan array screening of NY/107 carried out previously showed that this HA shows mixed binding to both avian and human receptors [20]. X-ray crystallography studies of NY/107 HA complexed with avian and human receptors have been solved. The binding of NY/107 HA to avian receptor is clearly observed in terms of resolving the coordinates of the sugar units in the RBS in the X-ray crystal structure [19]. On the other hand, much poorer electron density map and fewer interactions were observed for human receptor in RBS of NY/107 HA [19]. These structural observations do not fully explain the observed mixed binding to both avian and human receptors by this HA.

NY/107 HA showed predominant high affinity binding (Kd'∼63 pM) (Based on 3′SLN-LN) to avian receptors and a significantly lower binding to human receptors in our dose-dependent binding analysis (Figure 1C). The orders of magnitude higher relative affinity for binding to avian over human receptors by NY/107 HA is consistent with the observed interactions in the X-ray co-crystal structure of HA-glycan complexes [19]. Given that glycosylation at Asn-133 appeared to improve specificity for avian receptors in the H7N7 FC HA, we wanted to test if a similar effect was seen in the case of NY/107 HA specifically in the context of the deletion of the 220-loop. Therefore the Ala135→Thr mutation was introduced on NY/107 HA and this mutant HA showed the identical binding profile in a dose-dependent fashion as that of the wild type HA (Figure 1D). This result suggested that the glycosylation at Asn-133 is not likely to affect glycan-receptor binding of H7N2 HAs in which the 220-loop is deleted like in the case of NY/107 HA.

### Quantitative glycan-binding affinity of H7N7 HAs double Gln226→Leu/Gly228→Ser mutations

A double Gln226→Leu/Gly228→Ser mutations has quantitatively switched the glycan receptor binding specificity and affinity from avian receptor to human receptors for H3 and H2 HAs [12]. However such a double mutation has not quantitatively switched or increased binding of avian H5 HAs to human receptors [25], [26]. Given that H7 HA belongs to the same phylogenetic clade 2 as H3 HA, we wanted to evaluate the effect of the double mutation on the H7N7 HAs (given that NY/107 HA does not have the 220 loop).

**Figure 2:**
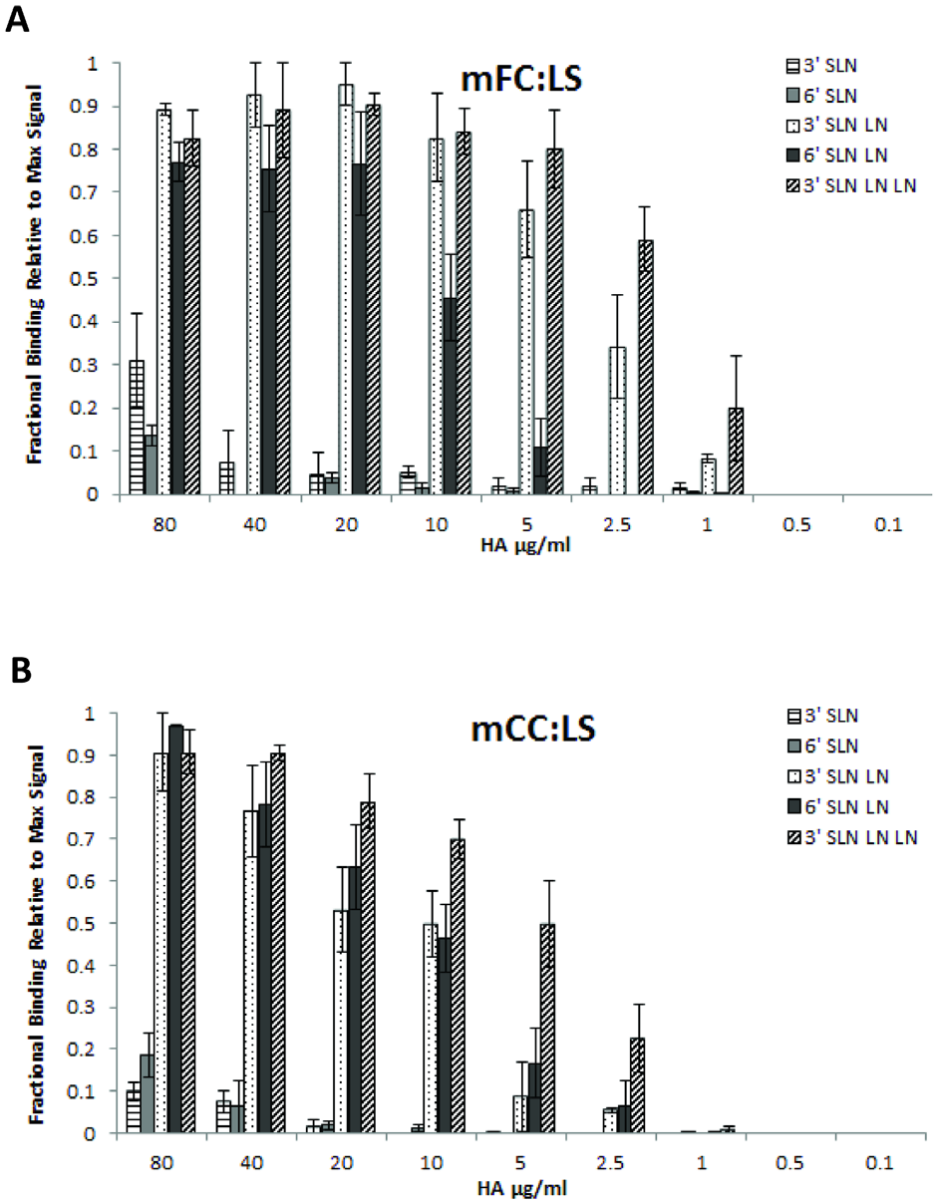
Glycan receptor-binding specificity of Gln226→Leu/Gly228→Ser mutant of FC and CC. **A and B**, respectively show dose-dependent direct glycan array binding of mFC: LS and mCC: LS mutant HAs. The double mutation leads to a substantial increase in human-receptor binding signals to a level that allowed calculation of apparent binding affinity parameter Kd'. The double mutation also lowers the avian-receptor binding affinity of mutant HAs relative to the corresponding wild-type HAs.

Introducing the double Gln226→Leu/Gly228→Ser mutations on FC (mFC:LS) and CC (mCC:LS) resulted in binding to both avian and human receptors. The human receptor binding affinity (Kd'∼1 nM) (Based on 6′ SLNLN binding) of mFC:LS and mCC:LS HAs was orders of magnitude higher (Figure 2A and 2B) and the human receptor binding observed for H7N7 CC and NY/107 HAs based on the quantitative dose-dependent binding assay. Interestingly the avian receptor binding affinity of mFC:LS (Kd∼70 pM) (based on 3′ SLN-LN) and mCC:LS (for Kd∼225 pM) (based on 3′SLN-LN) was lower than that of their respective wild type HAs (Figure 1A and 1B).

**Figure 3:**
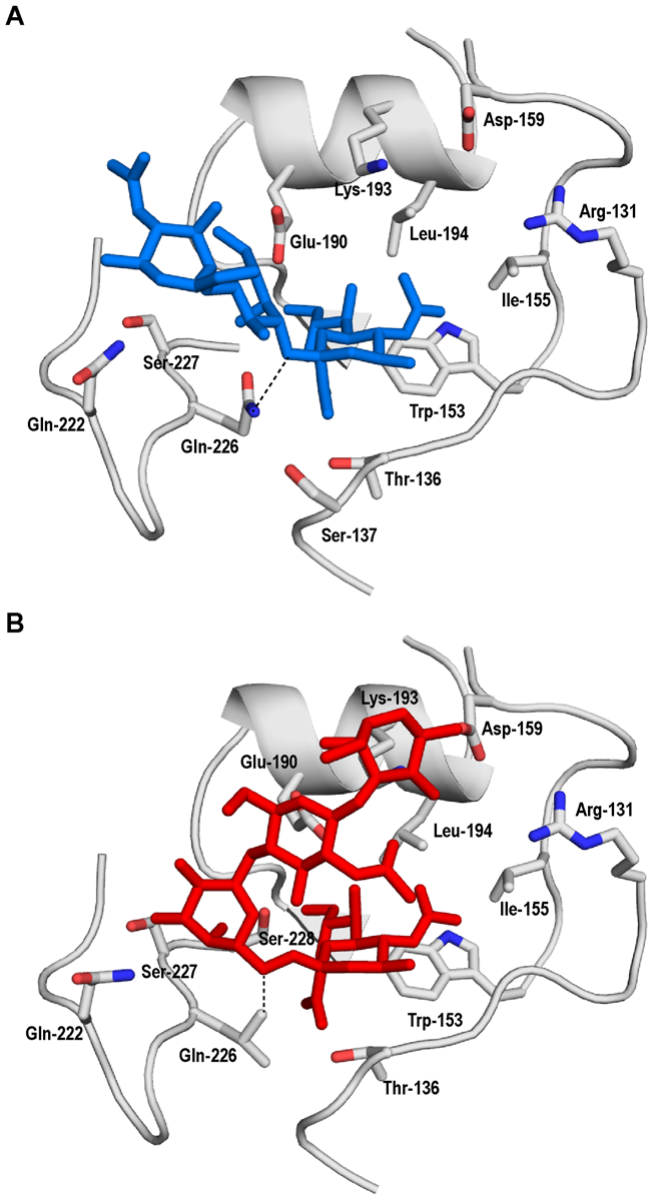
Structural complexes of FC – avian receptor and mFC:LS – human receptor. **A**, shows the structural complex of RBS of FC HA with avian receptor derived from the X-ray co-crystal structure of this complex (PDB ID: 4DJ7). **B**, shows structural complex of RBS of mFC:LS HA with human receptor derived based on homology-model of mFC:LS (generated using automated mode in Swiss Model: http://swissmodel.expasy.org/). The coordinates of the human receptor were obtained from PDB ID: 2WR7. The mFC:LS human receptor structural complex was obtained by superimposing the HA1 domain (bound to this receptor) of 2WR7 with that of mFC:LS HA1 homology model followed by subsequent energy minimization. The critical contacts made by Gln-226 (in FC HA) and Leu-226 (in mFC:LS HA) with glycan receptor is shown using a dotted line.

The crystal structure and co-crystal structures of FC in complex with human and avian receptor were solved recently [23]. These structures permitted comparison of molecular contacts of FC – avian receptor and mFC:LS – human receptor complexes (Figure 3). While Gln-226 of FC makes optimal contact with glycosidic oxygen atom of Neu5Acα2→Gal motif of the avian receptor, Leu-226 in the RBS of mFC:LS is positioned to make optimal contacts with C-6 atom of Gal sugar in the terminal Neu5Acα2→6Gal-motif of the human receptor. This observation offers an explanation the gain in human receptor binding of the double mutants. On the other hand Ser-228 of mFC:LS is positioned to make contact with both avian and human receptor. Therefore the loss in binding to avian receptor by Gln-226→Leu mutation is compensated in part by binding of Ser-228 to avian receptor. This structural observation is consistent with the observed avian receptor-binding of the double mutants.

### Binding of H7 HAs to human respiratory tissues

**Figure 4:**
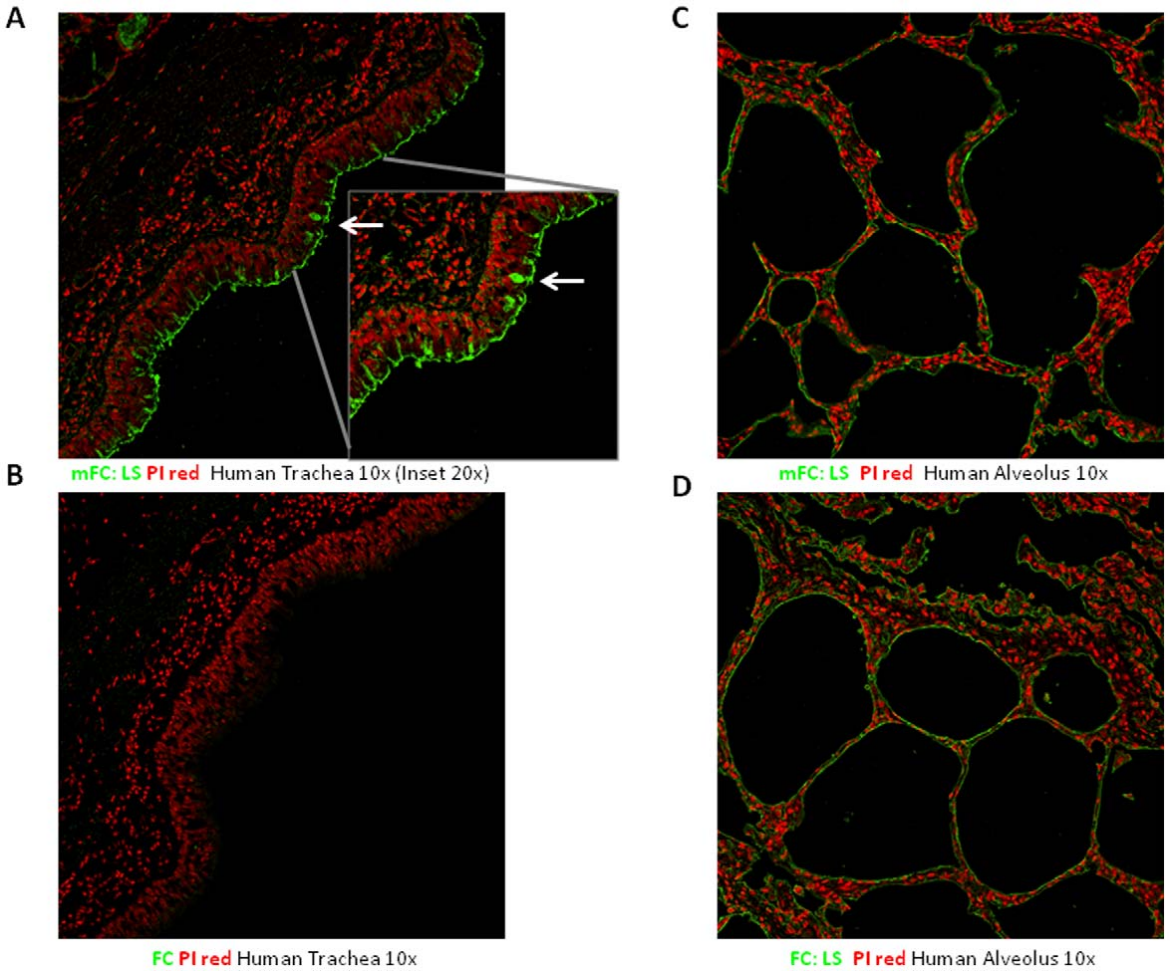
Tissue binding specificity of FC and mFC:LS for human tracheal and alveolar sections. **A**, Extensive staining of apical surface of human tracheal epithelia for the mFC: LS (green) against propidium iodide staining (in red) is observed. Bright staining of what appears to be goblet cells (inset at 20x magnification; indicated by *white arrow*) by this mutant HA resembles a similar pattern that was previously observed with 1918 H1N1 and 1958 H2N2 HAs. **B**, Shows minimal to no staining of apical surface of tracheal section by FC consistent with its low binding to human receptors on glycan array. **C** **and** **D** respectively show intense staining of alveolar section by mFC:LS and FC consistent with their high affinity binding to avian receptors.

To compare observed binding specificities of H7 HAs on the array with their binding to physiological glycan receptors, human tracheal (apical surface predominantly comprises of human receptors) and alveolar (predominantly comprises of avian receptors) sections were stained using representative wild type and the mutant HA with the double mutation. mFC: LS showed extensive staining of apical surface of the tracheal epithelium where human receptors are predominantly expressed (Figure 4A). Specifically it also predominantly stained what appear to be non-ciliated (goblet) cells. Extensive staining of goblet cells is a property that we have previously observed to be shared by human adapted HAs [12], [13], [27]. The staining of tracheal epithelium by the mutant HA is consistent with its observed human receptor-binding in the glycan array analysis. On the other hand, the wild-type FC HA showed minimal staining of the apical surface of the human tracheal epithelium (Figure 4B) consistent with its minimal human-receptor binding on the glycan array. Both the wild-type FC and mutant mFC:LS HAs showed extensive staining of the human alveolar section, which predominantly expresses α2→3 sialylated glycans (Figure 4C and 4D). This staining pattern is consistent with the binding of these HAs to the avian receptors on the glycan array.

## Discussion

The functions of HA in terms of glycan-receptor binding specificity and cleavage sequence for membrane fusion is among the key factors that contribute to the pathogenicity, severity of infection and transmissibility of influenza A virus. In this study we characterized in a quantitative fashion, the relative binding affinities of H7 HA to avian and human receptors given that some H7 strains especially from the Eurasian lineage are highly pathogenic and have caused human infections. To our knowledge, such a quantitative description of glycan-binding properties of H7 HA has not been reported earlier.

The FC, CC and NY/107 strains were also previously analyzed for their ability to transmit in the ferret animal model NY/107 and the highly pathogenic CC strain showed some transmission via direct contact, however the other highly pathogenic strain isolated from fatal case did not show any transmission. None of the viruses transmitted via respiratory droplets. Since we previously demonstrated that the human receptor-binding specificity and affinity correlates with respiratory droplet transmissibility in ferrets, we sought to investigate any potential mutations that would significantly increase the human-receptor binding of H7 HAs in this study. We demonstrated that the double Gln226→Leu/Gly228→Ser mutation (hallmark changes for human adaptation of H3 and H2 HA) dramatically increased human receptor-binding affinity of FC and CC HA. This study is therefore the first to report mutations in H7 HA that quantitatively increase its human receptor-binding affinity. Although none of the H7 HA sequences (obtained from NCBI flu database: http://www.ncbi.nlm.nih.gov/genomes/FLU/FLU.html) have Leu-226 and Ser-228, our study demonstrates that these mutations are easily accommodated in the H7 RBS to substantially increase binding to human receptors. Furthermore the Gln-226→Leu mutation has been observed in the H9 avian isolates. Therefore, it is important to monitor the evolution of current H7 HAs from the standpoint of their ability to acquire these mutations.

Although the double mutation increased human receptor binding of FC and CC HAs, the binding affinity of this HA to human receptor was still lower relative to avian receptor. This is not a typical characteristic of human-adapted HAs such as prototypic pandemic 1918 H1N1 and 1958 H2N2 HAs. However a natural variant of 1918 H1N1 HA isolated from humans which has a single amino acid mutation in the RBS (A/New York/1/18) shows a similar relative binding affinity as that of the mCC:LS and mFC: LS HAs. This observation warrants further investigation of the aerosol transmission in ferrets of reassorted viruses carrying these mutant H7 HAs in context of other human adapted genes similar to the previous studies carried out for H9 and H5 subtypes [7], [8].

In summary our results highlight the nuances in biochemical glycan-binding binding affinities of H7 HAs from two very different lineages and also show mutations in the Eurasian lineage that quantitatively increase their human receptor-binding affinity. Our studies would pave way for investigating the effect of these changes in contributing to the human adaptation of H7 HA based on additional ferret transmission studies that need to be performed on viruses carrying these mutant HAs.

## Materials and Methods

### Cloning, baculovirus synthesis, expression and purification of HA

Briefly, recombinant baculoviruses with FC or NY/107 gene and mutants off of each background, were used to infect (MOI = 1) suspension cultures of Sf9 cells (Invitrogen, Carlsbad, CA) cultured in BD Baculogold Max-XP SFM (BD Biosciences, San Jose, CA). The infection was monitored and the conditioned media was harvested 3–4 days post-infection. The soluble HA from the harvested conditioned media was purified using Nickel affinity chromatography (HisTrap HP columns, GE Healthcare, Piscataway, NJ). Eluting fractions containing HA were pooled, concentrated and buffer exchanged into 1X PBS pH 8.0 (Gibco) using 100K MWCO spin columns (Millipore, Billerica, MA). The purified protein was quantified using BCA method (Pierce). N-glycosylation is known to play an important role in folding and maintaining the three dimensional structure of HA [12]. In order to ascertain that mutation at position 143 did not affect protein stability circular dichroism analysis of the all wild type and mutant HAs was performed alongside H2 HA, A/Albany/6/58 (Alb58) isolated from the 1957–58 pandemic. The circular dichroism spectra of all the mutant proteins were generated between 190 nm and 280 nm. All the mutants showed similar circular dichroism spectral signatures as that of their wild-type counterparts and Alb58, a H2N2 HA (A/Albany/6/58) (**Figure S2**).

### Dose dependent direct binding of FC, NY/107 and mutant HAs

To investigate the multivalent HA-glycan interactions a streptavidin plate array comprising of representative biotinylated α2→3 and α2→6 sialylated glycans was used as described previously [13]. 3′SLN, 3′SLN-LN, 3′SLN-LN-LN are representative avian receptors. 6′SLN and 6′SLN-LN are representative human receptors (see **Table S1**). The biotinylated glycans were obtained from the Consortium of Functional Glycomics through their resource request program. Streptavidin-coated High Binding Capacity 384-well plates (Pierce) were loaded to the full capacity of each well by incubating the well with 50 µl of 2.4 µM of biotinylated glycans overnight at 4°C. Excess glycans were removed through extensive washing with PBS. The trimeric HA unit comprises of three HA monomers (and hence three RBS, one for each monomer). The spatial arrangement of the biotinylated glycans in the wells of the streptavidin plate array favors binding to only one of the three HA monomers in the trimeric HA unit. Therefore in order to specifically enhance the multivalency in the HA-glycan interactions, the recombinant HA proteins were pre-complexed with the primary and secondary antibodies in the molar ratio of 421 (HA: primary: secondary). The identical arrangement of 4 trimeric HA units in the pre-complex for all the HAs permit comparison between their glycan binding affinities. A stock solution containing appropriate amounts of Histidine tagged HA protein, primary antibody (Mouse anti 6X His tag IgG) and secondary antibody (HRP conjugated goat anti Mouse IgG (Santacruz Biotechnology) in the ratio 4∶2∶1 and incubated on ice for 20 min. Appropriate amounts of pre-complexed stock HA were diluted to 250 µl with 1% BSA in PBS. 50 µl of this pre-complexed HA was added to each of the glycan-coated wells and incubated at room temperature for 2 hours followed by the above wash steps. The binding signal was determined based on HRP activity using Amplex Red Peroxidase Assay (Invitrogen, CA) according to the manufacturer's instructions. The experiments were done in triplicate. Minimal binding signals were observed in the negative controls including binding of pre-complexed unit to wells without glycans and binding of the antibodies alone to the wells with glycans. The binding parameters, cooperativity (n) and apparent binding constant (Kd'), for HA-glycan binding were calculated by fitting the average binding signal value (from the triplicate analysis) and the HA concentration to the linearized form of the Hill equation:
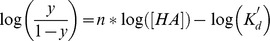
where y is the fractional saturation (average binding signal/maximum observed binding signal). In order to compare Kd' values, the values reported in this study correspond to the appropriate representative avian (3′SLN-LN or 3′SLN-LN-LN) and human (6′SLN-LN) receptor that gave the best fit to the above equation and the same slope value (n∼1.3).

### Binding of recombinant FC and mFC: LS HAs to human tracheal and alveolar tissue sections

Paraffinized human tracheal (US BioChain) tissue sections were deparaffinized, rehydrated and incubated with 1% BSA in PBS for 30 minutes to prevent non-specific binding. HA was pre-complexed with primary antibody (mouse anti 6X His tag, Abcam) and secondary antibody (Alexa fluor 488 goat anti mouse, Invitrogen) in a molar ratio of 4∶2∶1, respectively, for 20 minutes on ice. The tissue binding was performed over different HA concentrations by diluting the pre-complexed HA in 1% BSA-PBS. Tissue sections were then incubated with the HA-antibody complexes for 3 hours at RT. The tissue sections were counterstained by propidium iodide (Invitrogen; 1100 in TBST). The tissue sections were mounted and then viewed under a confocal microscope (Zeiss LSM 700 laser scanning confocal microscopy). Sialic-acid specific binding of HAs to tissue sections was confirmed by loss of staining after pre-treatment with Sialidase A (recombinant from *Arthrobacter ureafaciens*, Prozyme), This enzyme has been demonstrated to cleave the terminal Neu5Ac from both Neu5Acα2→3Gal and Neu5Acα2→6Gal motifs. In the case of sialidase pretreatment, tissue sections were incubated with 0.2 units of Sialidase A for 3 hours at 37°C prior to incubation with the proteins.

## Supporting Information

Figure S1 **Sequence Alignment of glycan-receptor binding site of H7 HAs.** Shown in the figure is the sequene alignment of HAs used in this study. The tk_Italy_H7N3 HA is also included since its X-ray crystal structure has been solved. The residue positions 133, 135, 226 and 228 are marked given that their properties have been modified throught mutagenesis in this study. The deletion of the 220-loop in NY/107 HA is also shown.
(PNG)

Figure S2 **Circular Dichroism Analysis of H7 wild type and mutant HAs used in the study.** Circular dichroism spectra for FC, CC, NY/107, mFC: Q226L, mCC: Q226L and Alb58 (A/Albany/6/58; H2N2 HA) are shown as indicated in the legend. All examined HAs show similar spectral signatures indicative of no general misfolding due to amino acid substitutions.
(TIF)

Table S1 **Expanded nomenclature of glycans used in the glycan array.** Table shows expanded nomenclature of the representative avian and human receptors used in glycan array. The monosaccharide key for the sugars is as follows – Neu5Ac: N-acetyl D-neuraminic acid; Gal: D-galactose; GlcNAc: N-acetyl D-glucosamine. α/β: anomeric configuration of the pyranose sugars. All the sugars are linked via a spacer to biotin (-Sp-LC-LC-Biotin as described in http://www.functionalglycomics.org/static/consortium/resources/resourcecored5.shtml).
(PDF)
