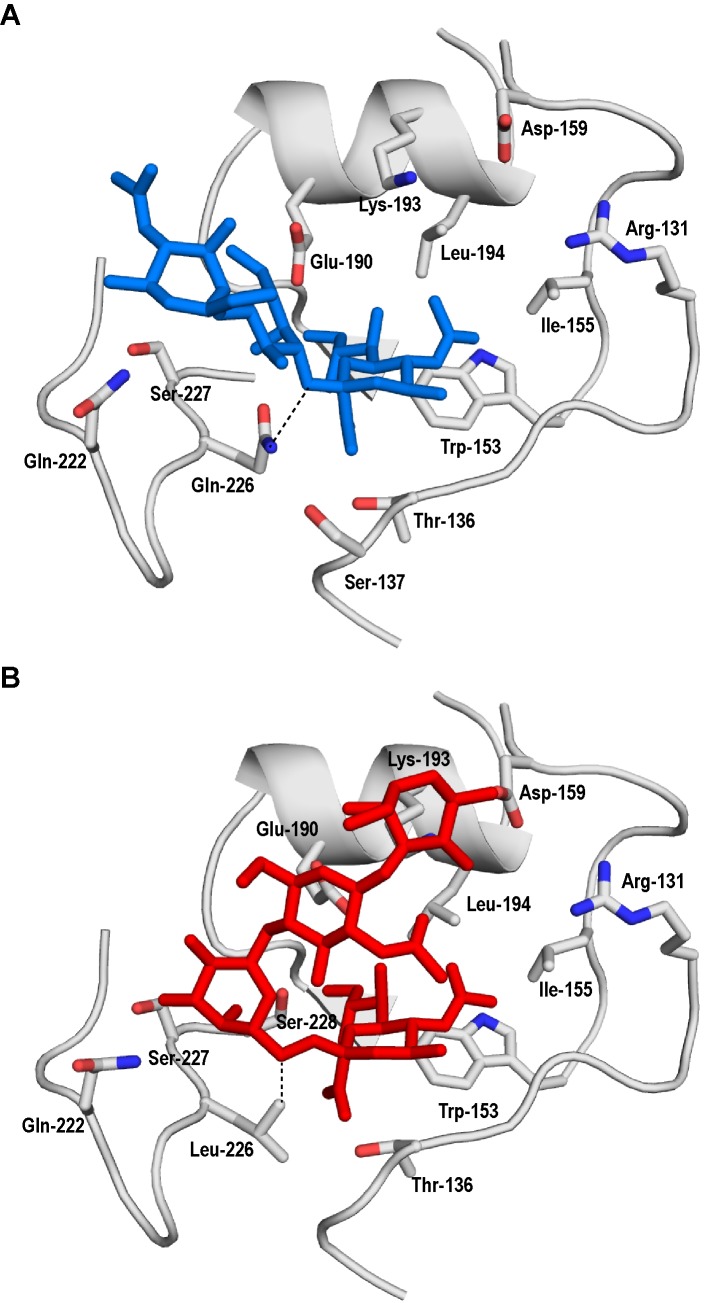# Correction: Quantitative Description of Glycan-Receptor Binding of Influenza A Virus H7 Hemagglutinin

**DOI:** 10.1371/annotation/c3b40a6f-a4be-4117-8ce2-a1b9b873e87c

**Published:** 2013-12-30

**Authors:** Karunya Srinivasan, Rahul Raman, Akila Jayaraman, Karthik Viswanathan, Ram Sasisekharan

Figure 3B is incorrect. Please see the correct Figure 3B here: 

**Figure:**