# Supplementary figures and images for: Quantitative Description of Glycan-Receptor Binding of Influenza A Virus H7 Hemagglutinin
Source: PLoS One. 2013 Feb 20;8(2):e49597. doi: 10.1371/journal.pone.0049597 (PMC3577880; doi:10.1371/journal.pone.0049597)

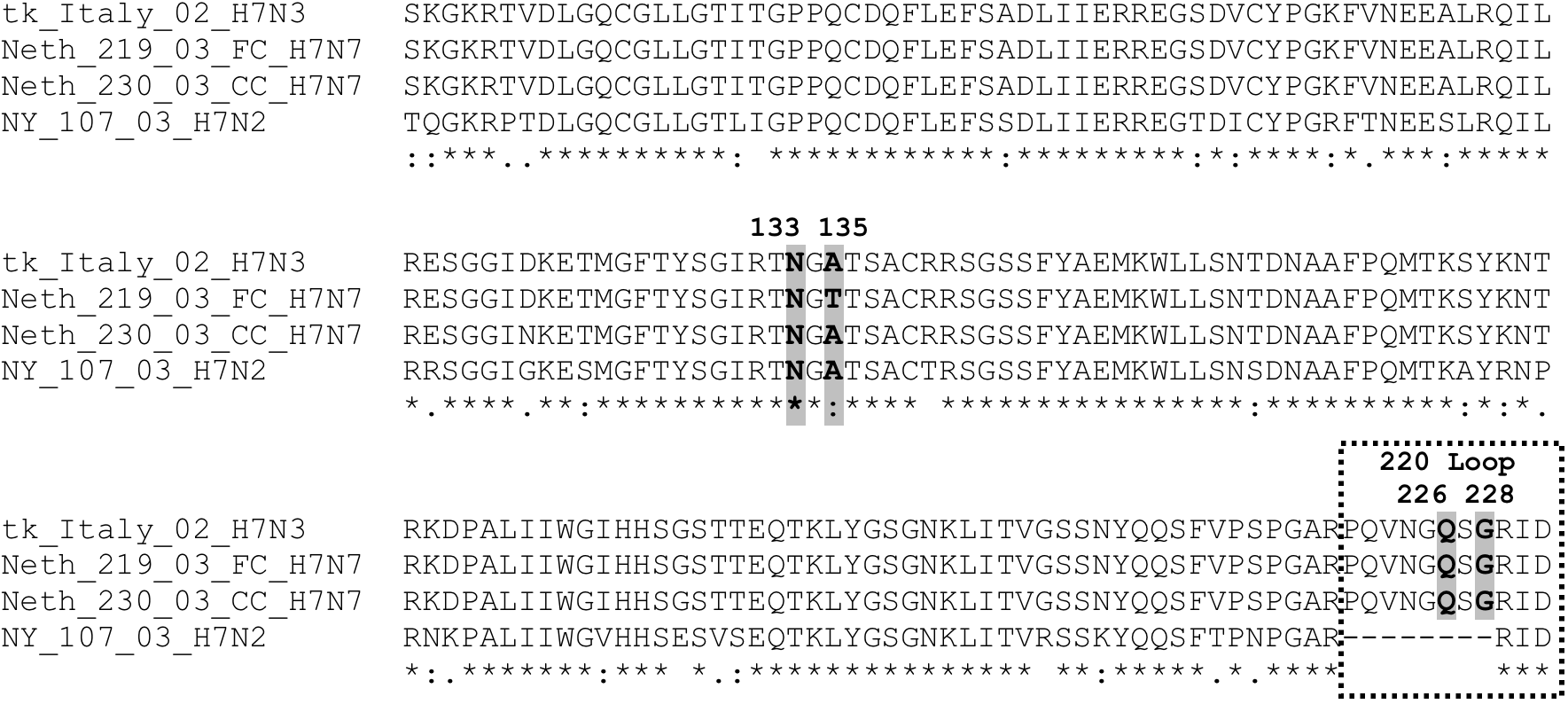

Supplement: Figure S1 — Sequence Alignment of glycan-receptor binding site of H7 HAs. Shown in the figure is the sequene alignment of HAs used in this study. The tk_Italy_H7N3 HA is also included since its X-ray crystal structure has been solved. The residue positions 133, 135, 226 and 228 are marked given that their properties have been modified throught mutagenesis in this study. The deletion of the 220-loop in NY/107 HA is also shown. (PNG) [file pone.0049597.s001.png]

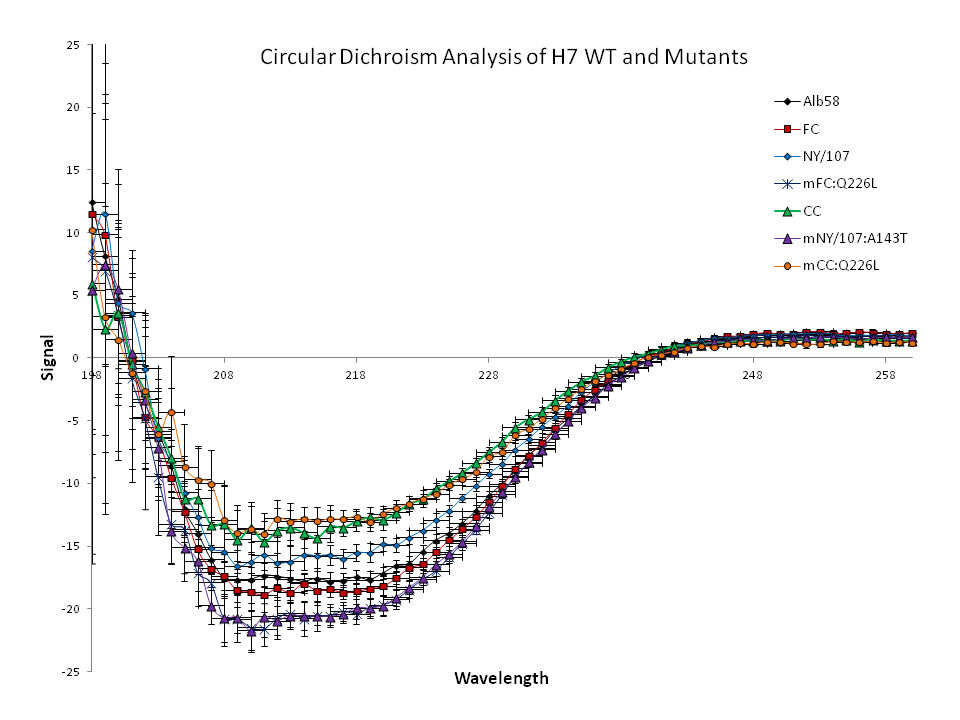

Supplement: Figure S2 — Circular Dichroism Analysis of H7 wild type and mutant HAs used in the study. Circular dichroism spectra for FC, CC, NY/107, mFC: Q226L, mCC: Q226L and Alb58 (A/Albany/6/58; H2N2 HA) are shown as indicated in the legend. All examined HAs show similar spectral signatures indicative of no general misfolding due to amino acid substitutions. (TIF) [file pone.0049597.s002.tif]
