# Supplementary material for: Quantitative Description of Glycan-Receptor Binding of Influenza A Virus H7 Hemagglutinin
Source: PLoS One. 2013 Feb 20;8(2):e49597. doi: 10.1371/journal.pone.0049597 (PMC3577880; doi:10.1371/journal.pone.0049597)
Supplement: Table S1 — Expanded nomenclature of glycans used in the glycan array. Table shows expanded nomenclature of the representative avian and human receptors used in glycan array. The monosaccharide key for the sugars is as follows – Neu5Ac: N-acetyl D-neuraminic acid; Gal: D-galactose; GlcNAc: N-acetyl D-glucosamine. α/β: anomeric configuration of the pyranose sugars. All the sugars are linked via a spacer to biotin (-Sp-LC-LC-Biotin as described in http://www.functionalglycomics.org/static/consortium/resources/resourcecored5.shtml). (PDF) [file pone.0049597.s003.pdf]

Supplementary Table S1. Expanded nomenclature of glycans used in the glycan array

**Glycan**	**Expanded nomenclature**
3'SLN	Neu5Acα2-3Galβ1-4GlcNAcβ1-
6'SLN	Neu5Acα2-6Galβ1-4GlcNAcβ1-
3'SLN-LN	Neu5Acα2-3Galβ1-4GlcNAcβ1-3Galβ1-4GlcNAcβ1-
6'SLN-LN	Neu5Acα2-6Galβ1-4GlcNAcβ1-3Galβ1-4GlcNAcβ1-
3'SLN-LN-LN	Neu5Acα2-3Galβ1-4GlcNAcβ1-3Galβ1-4GlcNAcβ1-3Galβ1-4GlcNAcβ1-

Key: Neu5Ac: N-acetyl D-neuraminic acid; Gal: D-galactose; GlcNAc: N-acetyl D-glucosamine. α / β: anomeric configuration of the pyranose sugars. All the sugars are linked via a spacer to biotin (-Sp-LC-LC-Biotin as described in http://www.functionalglycomics.org/static/consortium/resources/resourcecored5.shtml)
